# A Preliminary Assessment of Dispersion Level of SO_**2**_ in Fars Industrial Region, South of Iran, by GIS

**DOI:** 10.1155/2013/670590

**Published:** 2013-09-12

**Authors:** Mansooreh Dehghani, Mohammad Mehdi Taghizadeh, Hassan Hashemi, Ebrahim Rastgoo

**Affiliations:** ^1^Environmental Health Engineering Department, School of Health and Nutrition, Shiraz University of Medical Sciences, Shiraz, Iran; ^2^Environmental Engineering Department, Estahban Branch, Islamic Azad University, Estahban, Iran; ^3^Environment Research Center, Isfahan University of Medical Sciences, Isfahan, Iran; ^4^Apadana University, Shiraz, Iran

## Abstract

The city of Zarghan is located 25 km northeast of Shiraz, southern Iran. Zarghan is affected by numerous pollution sources such as oil refinery, an industrial park, and Shiraz-Tehran highway. The numerous contaminating sources around Zarghan can cause serious local air pollution. Sulfur dioxide gas is an important index of air pollution in cities. Therefore, in order to control and manage Zarghan air quality, it is important to monitor sulfur dioxide concentration in the surrounding area. It is also essential to know about the contribution level of other sources of pollution as well as dispersion radius of pollutants in the area. In this study, the concentration of sulfur dioxide was measured by passive sampling at 10 different stations. These values were interpolated in other parts of the city using ArcGIS software. The results of sampling showed that the concentration of the gas was 60 *µ*gm^−3^ around oil refinery. The level was 19 *µ*gm^−3^ in region located about 3 km from the oil refinery. It was also demonstrated that the gas concentration was not higher than the standard limit within residential area. On the other hand, the role of the local highway and industrial park was not significant in contaminating air in urban areas.

## 1. Introduction

Sulfur dioxide is one of the most important pollutants in urban areas which have caused numerous cases of respiratory diseases and deaths in many communities. Some studies show that there is a significant relationship between daily mortality and exposure to sulfur dioxide. Studies on asthma have not yet determined the safe limit of SO_2_ not causing respiratory malfunctioning. In fact, even very low concentrations of sulfur dioxide can cause respiratory capacity reduction [1, 2]. Maantay declared that people living in industrialized area of the Bronx and New York City were more susceptible for asthma (>66%) due to sulfur dioxide [3].

Previous studies have revealed that oil refineries were one of the major sources of emitting SO_2_ around and in the city [4]. Many other studies showed the effects of highways on dispersing SO_2_ around nearby cities [5]. Industrial parks could also be considered as other sources of the SO_2_ pollutant. The size of the industry, its distance from the neighboring town, and direction of the wind were among the most important parameters causing air pollution in cities. Many cities are being affected by a series of air pollution sources. Having a thorough knowledge on these sources is an important tool in controlling and managing them.

Geographic Information System (GIS) software is a powerful tool to determine the contribution levels of such sources. So far, many studies on controlling air pollution have been carried out using this software [6, 7]. In order to monitor geographical dispersion of a pollutant in an area, it is required that sample pollutant is to be examined at different stations simultaneously. Due to low cost and easy operation of diffusive method, this technique has been widely used to monitor air pollution with high precision in large scales. Diffusive sampling is a powerful tool to take samples of atmospheric gas using molecular diffusion with controlled speed that has no need to pump air during sampling [8]. Many studies on the accuracy of passive sampling of SO_2_ have shown that this method had an acceptable error rate compared to other methods of sampling [9]. Adame et al. [10] used K-mean cluster technique to measure daily cycles for SO_2_ pollutant at different air quality regimes in a heavily industrialized area in Spain. Four optimal cluster numbers were obtained for the daily patterns of SO_2_. While two of the clusters showed a low mixing ratio, the others exceeded the thresholds limits of air quality [10]. Bhanarkar et al. used a comprehensive emission inventory and dispersion modeling approaches to determine the contribution of SO_2_ from different types of pollution sources in Jamshedpur in India. Although the first approach showed that industrial sources account for 77% of the total emissions of SO_2_, the second approach demonstrated that more than 50% of SO_2_ was emitted from industrial sources [11]. Studying the effect of emission sources and meteorological conditions on SO_2_ pollution in Mongolia was done by Luvsan and his colleagues using multiple regression models. Data revealed that SO_2_ concentrations in industrialized area increased with the decrease of wind speed and temperature and with the increase of relative humidity [12]. SO_2_ distribution to air quality in Hong Kong was assessed by the multilayer, non-steady-state puff dispersion model. The results showed that the contribution of the power plant located in marine sources regions is significant during both summer and winter times [13].

Since the industrial regions of Zarghan (northeast of Shiraz) are affected by numerous air pollution sources, suitable monitoring systems are urgently needed that can rapidly and reliably detect and quantify polluting sources. Therefore, the main objectives of this study were to (i) determine preliminary assessment of the dispersion level of SO_2_ using passive sampling and GIS software and (ii) assess the contribution level of generating sources of SO_2_ in urban areas.

## 2. Materials and Methods

### 2.1. Zarghan Climatic Conditions

In order to study the dispersion level of SO_2_ in Zarghan, knowing the information about the local climatic condition is very essential. Zarghan average annual temperature is 16°C, and the total average annual rainfall is 330.2 mm. More than 56% of the precipitation falls during winter, and the number of rainy days is 48 days. The average annual humidity is 42%. Based on Domarten climate classification, the city is located on the semiarid area. Monthly average wind speed at the time of air sampling is 0.9 ms^−1^.

### 2.2. Locating Air Pollutant Sources in the Study Area

At first, the major sources of air pollution in the surrounding area of Zarghan are studied. Zarghan is located 25 km northeast of Shiraz, nearby Shiraz-Tehran highway. Many industrial complexes are located about 10 km from Zarghan among which one can name oil refinery, industrial park with more than 35 industries, ceramic industry, Loab industry, and chemical industry. Besides, due to a rocky wall of 300 m height in the east side of the town, the air flow through the town is often blocked. Each of the above sources individually could be the cause of air pollution in Zarghan.

### 2.3. Air Sampling for Detecting SO_2_

**Figure 1:**
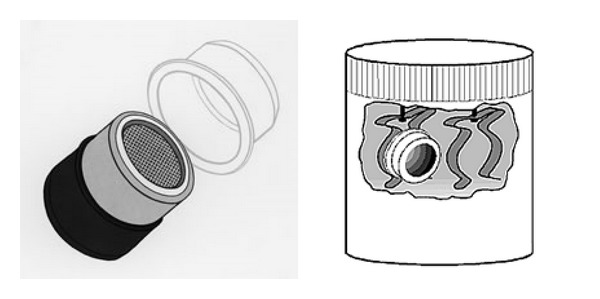
Clamp and SO_2_ adsorbent sample.

A diffusive sampler consisted of a tube in which the adsorbent material adsorbed the pollutant. Speed-controlled pollutant entered the adsorbent tube by molecular diffusion with no need for electrical power or pumping. Therefore, SO_2_ sampling was based on the adsorbent molecular diffusion. In this research, sampling tubes were sealed and carried to the lab for analysis of samples after two weeks of adsorption period. The tubes were of 20 mm diameters and made of polypropylene. To reduce the turbulence effects of the wind and rain, the sampling was taken by a metal clamp inside a fiberglass container as shown in Figure 1.

After collecting the specimens, they were sent to Pasam Company in Switzerland for the determination of SO_2_. Mixture of potassium carbonate and glycerin was used to extract SO_2_ and the total amount of extracted SO_2_ determined by ion chromatography.

### 2.4. SO_2_ Sample Collecting Locations

According to various sources, which are believed to be effective in Zarghan air pollution, boundary conditions for creating a mesh net and the locations of sampling were determined. Geographical coordinates, names, and reasons for choosing the sampling points are given in Table 1. Due to the small size of the study area, 10 points were selected and one sample was collected at each point. Moreover, it should be mentioned that statistical methods were not used in choosing the number of samples. Sampling adsorbents were installed by a crane at a height of 3 to 4 meters from the ground adjacent to electrical towers. The adsorbents were exposed to outdoor SO_2_ for 17 days from the second to the nineteenth of January, 2012.

### 2.5. Drawing GIS Maps

**Figure 2:**
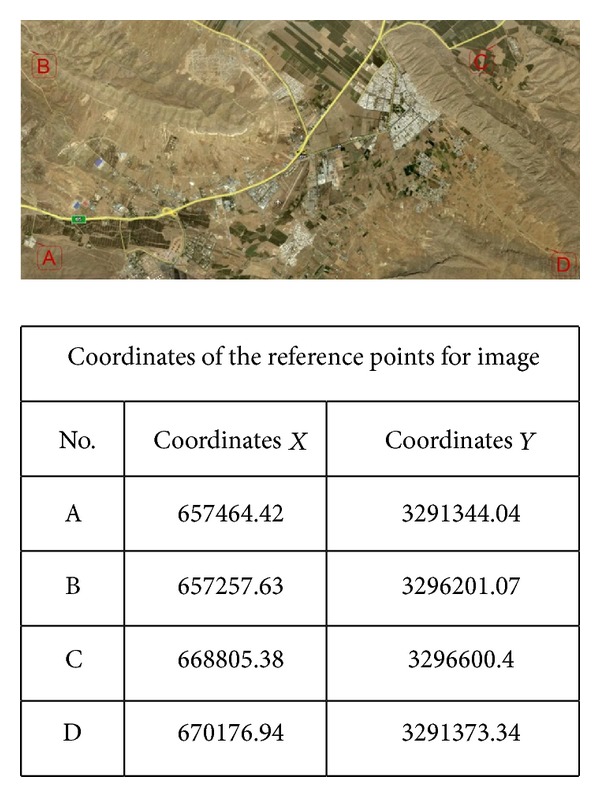
Coordinates of the reference points.

At the beginning, an image of the coverage area was obtained by Google Earth software. Geographical coordinates of 4 points of suitable dispersion were determined by the software and used in an Excel file as the ground reference (Figure 2).

Using ArcGIS software, the image was processed as the ground reference and the result saved in TIFF format with a pixel size of 5 m. The data obtained from sampling of SO_2_ were interpolated using the passive sampling method with different methods such as inverse distance weighing (IDW), natural nearest neighbor. Kriging format (TIFF) is a raster image format with a pixel size of 5 m for each specimen being prepared (with at least 10 sample points). Then, all interpolated layers were cut into the size of sample point's area to perform interpolation for all layers.

## 3. Results and Discussion

### 3.1. SO_2_ Passive Sampling

Table 2 shows the results of SO_2_ measurements in different stations in the industrial regions of Zarghan.

### 3.2. Regional Variation of SO_2_ Concentration Using GIS Software

**Figure 3:**
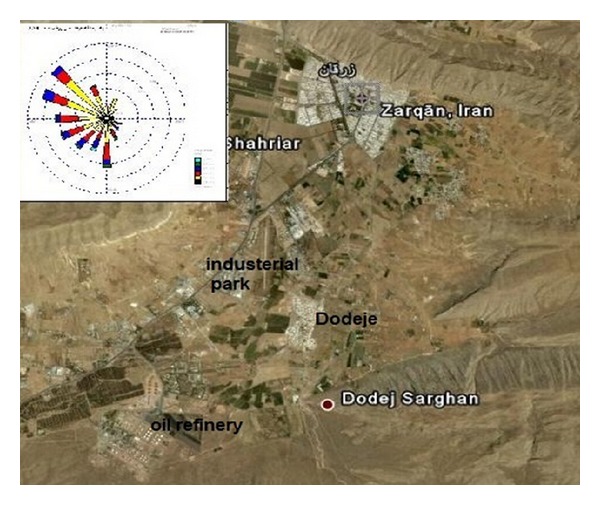
The maps of Zarghan and the wind rose of the sampling season.

**Figure 4:**
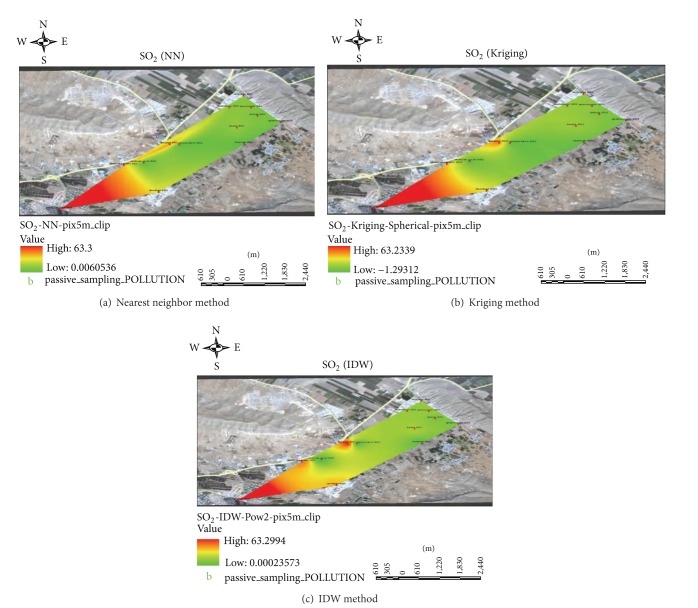
GIS results using different interpolation methods (nearest neighbor, Kriging, and IWD methods).

Due to the importance of wind direction, maps of the wind rose of the sampling season and Zarghan are shown in Figure 3. As shown in Figure 4, geographic concentration coverage area of SO_2_ has been studied using different methods of nearest neighbor, IWD, and Kriging. SO_2_ pollution maps were prepared using passive sample interpolation.

### 3.3. A Comparison of Measured SO_2_ Concentration with the Values Recommended by the WHO Air Quality Guidelines

WHO (World Health Organization) guideline value for 24 hours of SO_2_ concentration was set as 20 *µ*gm^−3^ [14]. Since in this study the passive sampling method was used and the specimens were exposed to the air pollution in many of the studied stations for 17 days, the data were comparable to the average 24-hour SO_2_ guideline. In general, many of the stations where samples were collected had acceptable SO_2_ levels except the following 4 stations which had considerable pollution levels.

1. The station of oil refinery with the value of 63.3 *µ*gm^−3^.
2. The station near street with the value of 29.7 *µ*gm^−3^.
3. The station of industrial park with the level of 24.4 *µ*gm^−3^.
4. The station with the level of 19.3 *µ*gm^−3^.

**Figure 5:**
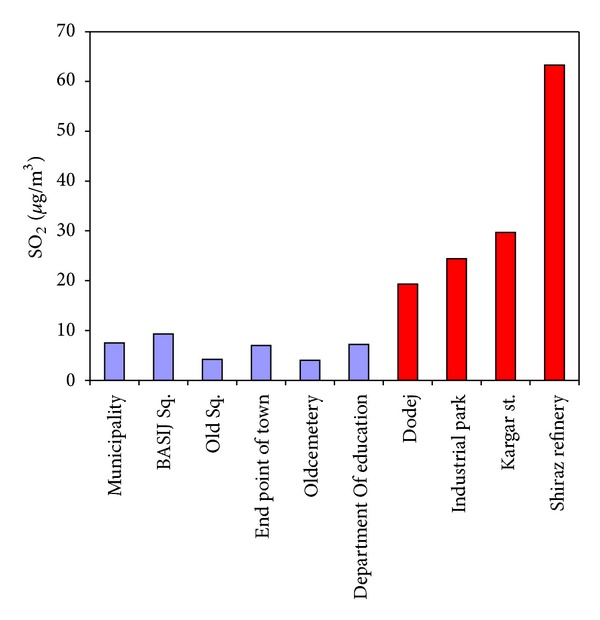
SO_2_ concentration in different stations in the industrial regions of Zarghan area.

Figure 5 shows SO_2_ concentration in different stations. As it can be seen in this figure, SO_2_ concentration in Zarghan residential areas is within standard limits.

### 3.4. GIS Data Analysis

As GIS pictures showed, among generating sources of SO_2_ pollution, the oil refinery and the highway were of more importance. In general, the concentration of SO_2_ was much greater around the oil refinery than near the highway. Although the prevailing wind direction at the time of sampling was not toward Zarghan, it could affect point located 3 km away. In addition the industries located in the industrial park could not have a major effect on SO_2_ concentration in Zarghan. The concentration of SO_2_ in the industrial park with 4000-meter distance from the-oil refinery was 24 *µ*gm^−3^ showing a higher concentration compared to point located 3 km away.

On the other hand, SO_2_ concentration at the distance of 200 meters from the highway inside the industrial park was greater than the one at station adjacent to the highway. It was concluded that the industrial park had intensified air pollution caused by the oil refinery by an amount of 5 *µ*gm^−3^. Although the prevailing wind in the highway blew toward Zarghan, it could have affected only 150 meters around. The 24-hour concentration of SO_2_ was just on standard border line in point located 3 km away, while it was within standard limits in Zarghan urban areas. The reason for higher level of SO_2_ in point located 3 km away was its adjacency to the oil refinery. 

According to the simulation of pollutants concentration in the atmosphere, using CALPUFF nonstationary Gaussian model at Rio Grande City, the concentration of CO reached 28.019 mg/m³, the SO_2_ reached 4.8623 mg/m³, and NO_2_ reached 40.8490 mg/m³ [15].

Similar study showed that most areas of Dallas County only experienced small variations of SO_2_ concentration over the period from 1996 to 2002 and that annual SO_2_ concentration in some areas of the county greatly increased over this period. In addition, results from the source-contribution analysis indicated that the spatial-temporal variations in SO_2_ concentration in Dallas County from 1996 to 2002 were not attributed to any particular type of emission source, but industrial emission sources could be the origin of extreme variations in SO_2_ concentrations in some areas [16]. 

Application of the SIM-air modeling tool in six Indian cities estimated that 21,400 premature deaths will be in the six cities in 2020; implementation of the six interventions in the transport and brick kiln sectors can potentially save 5870 lives (27%) annually and result in an annual reduction of 16.8 million tons of carbon dioxide emissions in the six cities [17].

As seen from the images, the nearest neighbor interpolation method showed the linear effects of pollution on the highway clearly. Pictures obtained by interpolation method revealed the expansion of pollutions in the area. 

## 4. Conclusions

As shown, Zarghan has been located in the green zone, and SO_2_ could not be accumulated in the town despite the existence of wind. According to the wind rose map, during January, the prevailing direction was not toward Zarghan. Therefore, it would be possible to record higher level of SO_2_ concentration in Zarghan if samples were collected during windy months blowing toward the city. On the contrary, the pollution due to the highway was affected by prevailing winds blowing toward the city, and, as seen in the GIS images, the highway pollution could not affect the city. It would be highly recommended to repeat the experiment during other months of the year, especially October, in which the prevailing wind direction is toward Zarghan.

## Figures and Tables

**Table 1:** Sampling locations in ten different stations in the industrial regions of Zarghan, Universal Transverse Mercator (UTM) coordinates, and reasons for the selection of that location.

No.	Sampling stations	UTM coordinates	Reasons for selection
1	Municipality	32995362.28MN 665921.42ME	Municipality location is a suitable point inside Zarghan residential area in mesh boundary.
2	Basij Sq.	3294970.72MN 666140.93ME	This point is a boundary residential location suitable for examining air pollution due to the SO_2_ of the highway.
3	Department of education	3295352.39MN 666550.56ME	This is one of the town central points near a hill and suitable to examine air inversion effects.
4	Old cemetery	3294683.63MN 667416.25ME	This point is located at southeast of the town on the border line near a hill.
5	Old Sq. (Emam Khomeini Sq.)	3294978.72MN 666740.94ME	Most of the air pollution in this area is due to heavy terrific jam than the other sources of contamination.
6	End point of town	3295906.31MN 666331.92ME	This residential point is located at the end of the town and the farthest to the oil refinery.
7	Near way Sq.	3291485.09MN 663854.37ME	This is the first urban area after oil refinery, located between Zarghan and the oil refinery.
8	Beginning of street	3293675.11MN 664173.69MN	This is a cross-point of the highway and Zarghan located 60 meters from Shiraz-Tehran highway.
9	Oil refinery	3295362.28MN 6659211.42ME	Oil refinery is a major source of SO_2_. Its distances from nearest residential points in towns are 6155 m and 2668 m, respectively.
10	Industrial park	3292829.20MN 6634148.2ME	This point was selected to examine the pollution roles of the oil refinery and industrial park.

**Table 2:** SO_2 _concentration (*µ*gm^−3^) in different sampling stations in the industrial regions of Zarghan.

No.	Sampling stations	SO_2_ concentration (*μ*gm^−3^)
1	Municipality	7.5
2	Basij Sq.	9.3
3	Department of education	7.2
4	Old cemetery	4.0
5	Old Sq. (Emam Khomeini Sq.)	4.2
6	End point of Town	7.0
7	Near way Sq.	19.3
8	Beginning of street	29.7
9	Oil refinery	63.3
10	Industrial park	24.4

## References

[1] Schwela D (2000). Air pollution and health in urban areas. *Reviews on Environmental Health*.

[2] Chen S-S, Tang C-S, Jin H-F, Du J-B (2011). Sulfur dioxide acts as a novel endogenous gaseous signaling molecule in the cardiovascular system. *Chinese Medical Journal*.

[3] Maantay J (2007). Asthma and air pollution in the Bronx: methodological and data considerations in using GIS for environmental justice and health research. *Health and Place*.

[4] Abed El-Raoof S (2009). Diurnal and seasonal variation of air pollution at Al-Hashimeya town, Jordan. *Jordan Journal of Earth and Environmental Sciences*.

[5] Brugge D, Durant JL, Rioux C (2007). Near-highway pollutants in motor vehicle exhaust: a review of epidemiologic evidence of cardiac and pulmonary health risks. *Environmental Health*.

[6] Matejicek L (2005). Spatial modelling of air pollution in urban areas with GIS: a case study on integrated database development. *Advances in Geosciences*.

[7] Pummakarnchana O, Tripathi N, Dutta J (2005). Air pollution monitoring and GIS modeling: a new use of nanotechnology based solid state gas sensors. *Science and Technology of Advanced Materials*.

[8] De Santis F, Fino A, Menichelli S, Vazzana C, Allegrini I (2004). Monitoring the air quality around an oil refinery through the use of diffusive sampling. *Analytical and Bioanalytical Chemistry*.

[9] Cruz LPS, Campos VP, Silva AMC, Tavares TM (2004). A field evaluation of a SO_2_ passive sampler in tropical industrial and urban air. *Atmospheric Environment*.

[10] Adame JA, Notario A, Villanueva F, Albaladejo J (2012). Application of cluster analysis to surface ozone, NO_2_ and SO_2_ daily patterns in an industrial area in Central-Southern Spain measured with a DOAS system. *Science of the Total Environment*.

[11] Bhanarkar AD, Goyal SK, Sivacoumar R, Chalapati Rao CV (2005). Assessment of contribution of SO_2_ and NO_2_ from different sources in Jamshedpur region, India. *Atmospheric Environment*.

[12] Luvsan ME, Shie RH, Purevdorj T, Badarch L, Baldorj B, Chan CC (2012). The influence of emission sources and meteorological conditions on SO_2_ pollution in Mongolia.. *Atmospheric Environment*.

[13] Yim SHL, Fung JCH, Lau AKH (2010). Use of high-resolution MM5/CALMET/CALPUFF system: SO_2_ apportionment to air quality in Hong Kong. *Atmospheric Environment*.

[14] World Health Organization (WHO) (2005). *Air Quality Guidelines: Global Update*.

[15] Thomasi CD, Nunes GAL, Jugueiro MM, Adamatti DF (2012). *GEO Processing: the 4th International Conference on Advanced Geographic Information Systems, Applications, and Services*.

[16] Zou B, Wilson JG, Zhan FB, Zeng Y, Wu K (2011). Spatial-temporal variations in regional ambient sulfur dioxide concentration and source-contribution analysis: a dispersion modeling approach. *Atmospheric Environment*.

[17] Guttikunda SK, Jawahar P (2012). Application of SIM-air modeling tools to assess air quality in Indian cities. *Atmospheric Environment*.

